# Building of Pressure-Assisted Ultra-High Temperature System and Its Inactivation of Bacterial Spores

**DOI:** 10.3389/fmicb.2019.01275

**Published:** 2019-06-10

**Authors:** Dong Liang, Liang Zhang, Xu Wang, Pan Wang, Xiaojun Liao, Xiaomeng Wu, Fang Chen, Xiaosong Hu

**Affiliations:** ^1^College of Food Science and Nutritional Engineering, National Engineering Research Centre for Fruits and Vegetables Processing, China Agricultural University, Beijing, China; ^2^Key Laboratory of Fruits and Vegetables Processing, Ministry of Agriculture, Beijing, China; ^3^College of Food Science, Northeast Agricultural University, Harbin, China

**Keywords:** pressure-assisted ultra-high temperature, mathematical model, bacterial spores, sterilization, inactivation mechanism

## Abstract

The pressure-assisted ultra-high temperature (PAUHT) system was built by using soybean oil as pressure-transmitting medium, and the multiple regression equation of soybean oil temperature change (Δ*T_P_*) during pressurization as a function of initial temperature (*T_i_*) and set pressure (*P*) was developed: Δ*T_P_* = -13.45 + 0.46 *T_i_* + 0.0799 P - 0.0037 $T_{i}^{2}$ - 2.83 × 10^-5^ P^2^. The fitted model indicated that the temperature of the system would achieve ≥121°C at 600 MPa when the initial temperature of soybean oil was ≥84°C. The PAUHT system could effectively inactivate spores of *Bacillus subtilis* 168 and *Clostridium sporogenes* PA3679 (less than 1 min). Treatment of 600 MPa and 121°C with no holding time resulted in a 6.75 log reductions of *B. subtilis* 168 spores, while treatment of 700 MPa and 121°C with pressure holding time of 20 s achieved more than 5 log reductions of *C. sporogenes* PA3679 spores. By comparing the PAUHT treatment with high pressure or thermal treatment alone, and also studying the effect of compression on spore inactivation during PAUHT treatment, the inactivation mechanism was further discussed and could be concluded as follows: both *B. subtilis* 168 and *C. sporogenes* PA3679 spores were triggered to germinate firstly by high pressure, which was enhanced by increased temperature, then the germinated spores were inactivated by heat.

## Introduction

Pressure assisted thermal sterilization (PATS), a technique combining high pressure with elevated temperature, has been used to effectively inactivate bacterial spores in low-acid food (Peleg et al., 2008; Nguyen et al., 2010; Olivier et al., 2011). Adiabatic compression caused by pressurization results in quick heating of the product to process temperatures, and the subsequent decompression results in its rapid cooling. Therefore, PATS can be conducted in a short time based on quick heating/cooling (Paidhungat et al., 2002; Ardia et al., 2004; Zhu et al., 2008; Reineke et al., 2013c). The overall treatment conditions are less severe than with conventional sterilization (Reineke et al., 2011; Doona et al., 2016a). All these attributes can lead to a better and healthier product, because unwanted reactions, like the Maillard reaction which are temperature and time dependent, might not occur or can be reduced by high pressure.

Generally, when the treatment was pressure of *P* = 600–800 MPa and initial temperature of *T_i_* = 90–120°C, all spores could be inactivated (Margosch et al., 2006; Wilson et al., 2008; Lopes et al., 2018). However, studies on PATS usually use water as pressure-transmitting medium and the final temperature is usually less than 121°C after compression heating, and it still requires several minutes (> 3 min) to inactivate all spores (Margosch et al., 2004b; Ramaswamy and Balasubramaniam, 2007; Shao and Ramaswamy, 2008; Doona et al., 2017). In order to achieve higher ration of spore inactivation in shorter time, higher pressure and temperature could be a good choice for the PATS processing (Shao and Ramaswamy, 2008; Wang et al., 2017). Nowadays, studies on spore inactivation by PATS with final temperature more than 121°C are relatively limited. In order to achieve higher temperature during PATS treatment, we supposed that the soybean oil could be used as the pressure-transmitting medium because of its higher compression heating coefficient (9.2°C/100 MPa) (Patazca et al., 2007; Zhu et al., 2008). This PATS system with higher working temperature could be defined as pressure-assistant ultra-high temperature (PAUHT) system. This system is rarely built and its inactivation effect and mechanism of bacterial spores has never been investigated.

The mechanism of spore inactivation by PATS has been extensively studied (Mathys et al., 2009; Ramaswamy et al., 2013; Reddy et al., 2016). Generally, it is proposed that spores are firstly induced to germinate and lose resistance, and then inactivated by heat. However, spore germination induced by PATS is different due to different treatment conditions and species of spores. For spores of *Bacillus* species, the germination is different between moderate pressure (200–500 MPa) and higher pressure (> 500 MPa) (Reineke et al., 2013b). At moderate pressure, spores are induced to germinate by activating the germinant receptors (GRs) and ensuing the release of DPA (Paidhungat et al., 2002; Setlow, 2003; Vepachedu and Setlow, 2007; Doona et al., 2014). DPA release activates one of the cortex lytic enzymes (CLEs), CwlJ, and induces spore core partial hydration, which may activate another CLEs, SleB (Setlow et al., 2009; Paredes-Sabja et al., 2011). These two activated CLEs degrade the cortex, resulting in completion of spore germination (Li et al., 2013). However, the higher pressure (> 500 MPa) induced germination was triggered by directly opening the DPA channel, SpoVA, without activating the GRs (Elke and Wuytack, 2001; Vercammen et al., 2012; Reineke et al., 2013c; Sevenich et al., 2013; Kong et al., 2014; Sarker et al., 2015). Margosch and others proposed a spore inactivation mechanism by PATS that is not involved spore germination. These researchers suggest that the inactivation mechanism of *B. subtilis* and *Bacillus licheniformis* spores by PATS at temperature higher than 70°C follows a two-stage strategy: (i) DPA is released by a short HHP pulse at high temperature and (ii) then spores are thermally inactivated-independent of pressure level upon depressurization (Margosch et al., 2004a). For *Clostridial* spores, Doona and others reported *Clostridium perfringens* spores with GRs exhibit similar germination-inactivation mechanism to spores of *Bacillus* species when treated by PATS (Doona et al., 2016a,b). However, for *Clostridium difficile* spores without GRs, spores were not germinated by pressure of 150 MPa. In contrast, the pressure of 550 MPa induced spores to release DPA, but the spores did not complete germination and remained heat resistant. As for *C. sporogenes* spores with GRs similar to *C. perfringens* spores (Setlow et al., 2017), they can theoretically germinate during PATS treatment and lose heat resistance, but it has never been verified.

The objectives of this study were to (1) build the PAUHT system with soybean oil as pressure-transmitting medium, (2) investigate the inactivation of PAUHT on *B. subtilis* 168 and *C. sporogenes* PA3679 spores, and (3) study the inactivation mechanism of spores under the condition of PAUHT.

## Materials and Methods

### Bacterial Strains and Preparation of Spore Suspensions

The bacterial strains used in the study were *B. subtilis* 168, FB85 (without GRs, a derivative of strain 168) (Paidhungat and Setlow, 2000) and *C. sporogenes* PA3679. The *B. subtilis* 168 and *C. sporogenes* PA3679 were obtained from China General Microbiology Culture Collection Center and China Center of Industrial Culture Collection, respectively. FB85 was a gift from Prof. Peter Setlow. *B. subtilis* 168 and FB85 strains were grown in Luria Bertani broth medium. Spores were prepared at 37°C by plating aliquots of 0.2 mL from the fresh overnight culture on the 2 × Schaeffer’s glucose medium agar plates without antibiotics as described previously (Paidhungat and Setlow, 2000; Luu et al., 2015). The spores were harvested after 2 days of incubation when over 90% of the spores were released from the mother cell as observed by phase contrast microscopy (Axio Observer. A1, Carl Zeiss, Germany). The spore suspension was cleaned by repeated centrifugation (8000 × *g*, 10 min, 4°C) for at least five times with cold distilled water (4°C). Then, the spores were cleaned using the histodenz gradient centrifugation and washed three times with sterilized water. Finally, the spore suspensions were stored in the dark at 4°C and were 98% free of growing or sporulating cells, germinated spores, and cell debris as shown in Supplementary Figure S1 observed by phase-contrast microscope (Setlow, 2018).

*Clostridium sporogenes* PA3679 was grown anaerobically in the Reinforce Clostridial Medium (RCM) broth in anaerobic jars with an Oxoid GasPak sachet (Oxoid, Basingstoke, United Kingdom) for 2 days at 37°C. An aliquot (0.2 mL) of *C. sporogenes* PA3679 culture was spread on the RCM plate and incubated at 37°C anaerobically for more than 5 days. The spores were harvested when over 90% of the spores were released from the mother cell observed by phase contrast microscopy. Then, the spore suspension was cleaned by centrifugation same as spores of *B. subtilis* strains. Finally, the spores were suspended in the sterilized water and stored at 4°C for the subsequently use. The concentration of the spore suspensions was adjusted to approximate 10^8^ CFU/mL before treatments.

### High Pressure Equipment

**FIGURE 1:**
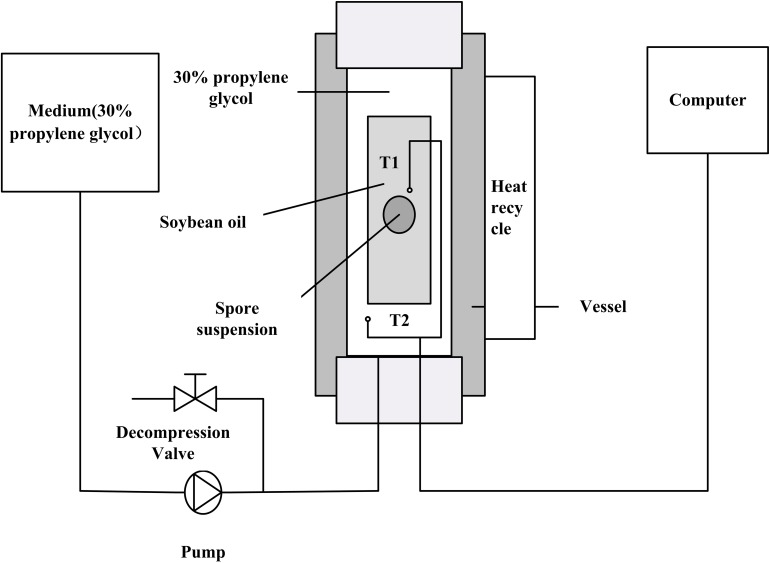
Schematic diagram of the high-pressure experimental system: T_1_ and T_2_ are thermocouples in soybean oil insulator medium and high hydrostatic pressure vessel medium, respectively.

High pressure treatment was carried out with an experimental setup as show in Figure 1. The HP device (FPG 7100, Stansted Fluid Power Ltd., Essex, United Kingdom) had a 2 L cylindrical pressure chamber (70 mm in diameter and 500 mm in height) with a maximum working pressure of 900 MPa. The pressure chamber was surrounded by a heating sleeve and the maximum temperature was 110°C. A data logger connected with T-type thermocouples (Omega Engineering, CT, United States) was used to gather the *in situ* pressure and temperature data. The medium used for pressure transmission in the system was 30% propylene glycol.

### HHP Treatment

A polyoxymethylene (POM) plastic insulated chamber (18 mm inner diameter, 50 mm outer diameter, and 200 mm height) was used to hold soybean oil and the sample during the pressure treatment to provide the temperature stability as described elsewhere (Shao and Ramaswamy, 2008). The soybean oil was filled into the insulator and used as the pressurization medium to get desired temperature by compression heating.

Two mL of spore suspensions were packed in the sterilized polyamides/polyethylene plastic bags (4 cm × 4 cm) by heat sealer. The insulator and spore sample were preheated to the initial temperature prior HHP treatment. HHP treatment was carried out immediately after the spore was placed into the insulator. The compression rate was ∼350 MPa/min, and the depressurization time was less than 5 s at all pressure levels. Different pressure levels (600 and 700 MPa) combined with temperatures (121, 125, and 130°C) were set for the treatments. The treated samples were then cooled in an ice bath and immediately analyzed.

### Thermal Treatment

Capillary tubes (0.9 mm inner diameter, 1 mm diameter, and 100 mm length) were used for the thermal treatments at 121, 125, and 130°C in the methyl silicone oil bath by a heating circulator. Twenty μL of spore suspension was injected into the tubes by micropipette and sealed by flame as described elsewhere (Sapru et al., 1992). After heating, capillary tubes were cooled immediately in ice water, cleaned with 70% ethyl alcohol for 10 min, and washed with sterilized water. The spore suspension was flushed out with a pipette and analyzed.

### The Measurement of DPA Release

After different treatments, the DPA release was determined by measuring the fluorescence of DPA with Tb^3+^ using a multi-well 96 fluorescence plate reader (Spark 10 M, Tecan) as described elsewhere (Yi and Setlow, 2010). After treatment, 50 μL spore suspension were mixed in the HEPES-TbCl_3_ solution, which consisted of 50 mM HEPES (pH 7.4) buffer and 200 μM TbCl_3_, the volume was made to 200 μL. Analytic grade water was used for the preparation of the buffer solution. DPA release was determined by the measurement of fluorescence emission at 545 nm with excitation at 270 nm. The maximum amount of DPA in the spore suspensions was determined by a thermal treatment for 20 min at 121°C.

### Determination of Cell Count

*Bacillus subtilis* and *C. sporogenes* spore counts were determined by pour-plate enumeration in duplicate, using the nutrient agar and RCM agar, respectively. One milliliter dilution of spore suspension was added into each plate. The *B. subtilis* plate was incubated aerobically at 37°C for 24 h, while the *C*. *sporogenes* plate was incubated anaerobically at 37°C for 36 h before enumeration. The untreated samples were used as a control for each experiment in order to obtain the initial spore counts. The logarithm of survivors [log_10_ (*N*_0_/*N*_t_)] was used as the spore reduction after different treatments. *N*_0_ and *N*_t_ were the spore counts before and after treatment, respectively. The initial concentration of the spore suspensions was adjusted to approximate 10^8^ CFU/mL before treatments.

### Microscope Analysis

After different treatments, the spore suspensions (OD_600_ = 1.0) were double stained with 0.5 μM SYTO16 (Invitrogen, Carlsbad, CA, United States) and 15 μM propidium iodide (PI) (Invitrogen, Carlsbad, CA, United States). Suspensions were vigorously mixed and incubated in the dark for 15 min prior to analysis as described previously (Reineke et al., 2013a). Both fluorescent dyes are able to stain DNA, the membrane permeant SYTO 16 acts as an indicator for spore germination, whereas the membrane impermeant PI indicates membrane damage (Mathys et al., 2009). The spores were observed by phase contrast and fluorescence microscopy (Axio Observer. A1, Carl Zeiss, Germany).

### Statistical Analysis

All experiments were carried out in triplicate and data were presented as mean ± standard deviation. The one-way analysis of variance was used to test the statistically significant differences (*P* < 0.05) between treatments using software SPSS 17 for windows (SPSS Statistical Software, Inc., Chicago, IL, United States). Software Origin 7.5 (Origin Lab, MA, United States) was used for making plots and fitting mathematical model.

## Results and Discussion

### The Pressure-Temperature Profile of the PAUHT System

**FIGURE 2:**
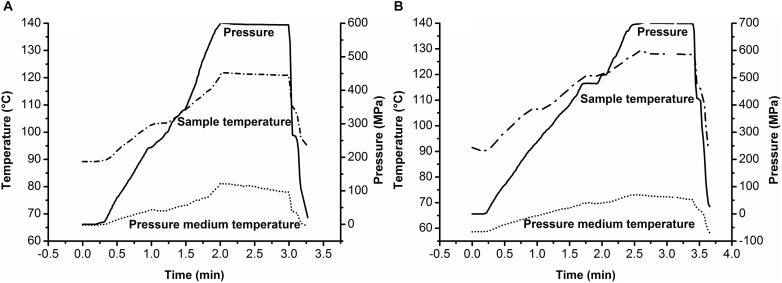
Typical pressure–temperature profiles of soybean oil during PAUHT treatments: **(A)** 600 MPa and 121°C (1 min), and **(B)** 700 MPa and 130°C (1 min).

Under compression during HHP treatment, the increased internal energy of the system resulted in a rapid rise in temperature (Ramaswamy and Balasubramaniam, 2007; Shao et al., 2010). In this PAUHT system, soybean oil was used as the pressure-transmitting medium to get the desired ultra-high temperature by increasing the initial temperature with compression heating. Figure 2 showed a typical temperature change of the soybean oil with different initial temperature during PAUHT treatment. When the initial temperature of soybean oil was set at 86°C, the final temperature reached 121°C with pressure of 600 MPa (Figure 2A). Under 700 MPa (Figure 2B), the final temperature of the medium reached to 130°C when the initial temperature was set at 90°C. The temperature increase (30–40°C) of soybean oil during PAUHT treatment was much more than that (11–16°C) of the 30% propylene glycol, indicating that the soybean oil has a better compression heating behavior than the 30% propylene glycol, which was consistent with the results previously reported (Houška et al., 2004; Min et al., 2010). Moreover, during the pressure holding time, the temperature of soybean oil remains almost unchanged in the insulator, indicating the POM material can be used for the insulator to remain the temperature stable. All of the results above suggested that the PAUHT constructed with soybean oil as pressure-transmitting medium has the capability to reach ultra-high temperature up to 130°C with excellent stability during the process, hence it could be used as a reliable tool for the follow-up studies.

In order to predict the temperature change (Δ*T_P_*) of soybean oil during PAUHT treatment, 100 data points from the measurements were pooled to develop a multiple regression equation of Δ*T_P_* of soybean oil during pressurization as a function of initial temperature (*T_i_*) of soybean oil and set pressure (*P*). It could be expressed as a quadratic function of *P* and *T_i_* as follows:

(1) $$\Delta T_{P}=-14.09+0.47\;T_{i}+0.082\;P-3.1\times 10^{-5}\;T_{i}P-0.004\;T_{i}^{2}-2.8\times 10^{-5}\;P^{2}$$

(*R*^2^_adj_ = 0.993, *p* < 0.05 for all items).

Where *T_i_* = initial temperature (50°C < *T_i_* < 90°C), *P* = pressure (100 MPa < *P* < 700 MPa), *T_p_* = target temperature (°C), Δ*T_P_* = temperature change (°C), *R*^2^_adj_ = adjusted *R* square, *p* = statistic parameter of significance.

When desired PAUHT conditions were given, the initial temperature of soybean oil could also be calculated by the following equation:

(2) $$T_{i}=18.22+0.44\;T_{P}-0.05\;P-4.61\times 10^{-4}\;T_{P}P+0.0042\;T_{P}^{2}+4.25\times 10^{-5}\;P^{2}$$

(*R*^2^_adj_ = 0.993, *p* < 0.05 for all items).

The parameters of these equations were determined by enter regression procedure. The adjusted regression coefficient (*R^2^*_adj_) value was used to measure the performance of the proposed model. Eq. (2) can be used to calculate the initial temperature under different combinations of *T_p_* and *P*-values.

The used conditions were listed in Table 1. The high temperature of 121°C can be achieved under PAUHT at 600 MPa commonly used in industry, as long as the initial temperature of soybean oil was 84°C. The verification experiment was done to test the proposed model. When the initial temperature was 84°C at 600 MPa, the final temperature of soybean oil can reach to 120.86°C, which was within the range of the confidence interval. As a result, this equation can be used to predict the initial temperature of soybean oil for the PAUHT treatments.

**Table 1:** Experimental setup and temperature control for PAUHT treatments.

System pressure (MPa)	Target final temperature (°C)	Required initial temperature (°C)	Achieved final temperature (°C)
600	121	84	120.86
	125	89	124.89
	130	95	130.37
700	121	79	121.05
	125	84	124.69
	130	90	129.73


### The Effect of PAUHT on Spore Inactivation

The inactivation of *B. subtilis* 168 and *C. sporogenes* PA3679 spores under different PAUHT treatments were shown in Table 2. The treatment of 600 MPa at 121°C without pressure holding time resulted in 6.75 log and 2.56 log reductions of *B. subtilis* 168 and *C. sporogenes* PA 3679 spores, respectively, indicating that spores of *C*. *sporogenes* PA 3679 were more resistant to pressure and temperature than spores of *B. subtilis* 168, as reported previously (Wilson et al., 2008; Ramaswamy et al., 2013). Moreover, when the temperature increased to 125°C or hold time up to 1 min, the amount of *B. subtilis* 168 spores was below the detection line, and the inactivation of *C. sporogenes* PA3679 spores was also increased. These results indicated the PAUHT treatments could effectively inactivate *B. subtilis* 168 and *C. sporogenes* PA3679 spores with less than 1 min of holding time. During pressure holding time for 1 min, the treatment of 600 MPa at 121°C resulted in 3.58 log reductions of *C. sporogenes* PA3679 spores. Increasing the pressure to 700 MPa, the treatment at 121°C for 1 min of holding time resulted in 6.31 log destructions of *C. sporogenes* PA3679 spores. Moreover, increasing the temperature to 125°C, the treatment of 600 MPa for 1 min of holding time induced 8.92 log *C. sporogenes* PA3679 spore inactivation. These results indicated that increase of pressure and temperature significantly accelerated the spore inactivation during pressure holding time of PAUHT treatments, which was consistent with previous report (Ahn et al., 2007). In addition, during PAUHT treatment, a compression of 120–140 s (∼5 MPa/s) was needed to arrive the final pressure of *P* = 600–700 MPa (Figure 2). Even without pressure holding time, 6.75 log and 2.56 log reductions of *B. subtilis* 168 and *C. sporogenes* PA3679 spores were achieved at 121°C and 600 MPa, respectively. Moreover, the treatment of 700 MPa at 121°C without pressure holding time resulted in 8.85 log (under detection line) and 4.53 log destructions of *B. subtilis* 168 and *C. sporogenes* PA3679 spores, respectively (Table 2). These results indicated that the compression played an important role in spore inactivation and should be taken into consideration during PAUHT treatments. Notably, the treatment of 700 MPa for 20 s at 121°C resulted in more than 5 log inactivation of *C. sporogenes* PA3679 spores. A 5 log reduction is regarded as the commercial sterilization standard for *C. sporogenes* PA3679 spores by food industry and regulatory agencies due to the fact that *C. sporogenes* spores are more heat resistant than *C. botulinum* spores (USFDA-FSIS, 2005; Ramaswamy et al., 2013; Reddy et al., 2016). Moreover, thermal sterilization required a process at 121.1°C for over 2.52 min to reach the goal of commercial sterilization (Theron and Irving, 1977), while using the PAUHT processing (700 MPa, 121°C) proposed here a processing time of 20 s was needed for the same effect, thus the PAUHT processing was more time efficient than thermal processing. However, although PAUHT treatment at temperature higher than 121°C could be more efficient at inactivation spores (Table 2, 600 MPa/125°C/0 s, 700 MPa/130°C/0 s), this higher temperatures would also produce unwanted side-reactions and degrades food quality (Lau and Turek, 2007). Hence, the following studies were mainly focused on the PAUHT treatment at 121°C.

**Table 2:** The inactivation and DPA release of *B. subtilis* 168 and *C. sporogenes* PA 3679 spores after different treatments.

Pressure, temperature, pressure holding time	*B. subtilis* 168		*C. sporogenes* PA3679	
		
	-Log (*N*_t_/*N*_0_)	DPA release (%)	-Log (*N*_t_/*N*_0_)	DPA release (%)
600 MPa, 121°C, 0 s	6.75 ± 0.32	95.15	2.56 ± 0.15	90.13
600 MPa, 121°C, 1 min	–	96.53	3.58 ± 0.33	92.85
600 MPa, 125°C, 0 s	–	96.46	5.43 ± 0.32	95.79
600 MPa, 125°C, 20 s	–	97.32	7.21 ± 0.41	98.78
600 MPa, 125°C, 1 min	–	98.31	–	98.89
700 MPa, 121°C, 0 s	–	98.72	4.53 ± 0.65	97.05
700 MPa, 121°C, 20 s	–	98.79	5.01 ± 0.22	97.88
700 MPa, 121°C, 1 min	–	98.88	6.31 ± 0.32	98.28
700 MPa, 130°C, 0 s	–	98.69	7.13 ± 0.11	98.66
700 MPa, 130°C, 10 s	–	98.88	–	98.83
700 MPa, 130°C, 20 s	–	98.63	–	98.78


*The initial total colony of *B. subtilis* spores was: 7.15 × 10^*8*^ CFU/mL; the initial total colony of *C. sporogenes* spores was: 8.23 × 10^*8*^ CFU/mL. 0 s represents decompression immediately after pressurization. “-” Represents spore counts were under the detection limit.*

### The Inactivation Mechanism of Spore by PAUHT

**FIGURE 3:**
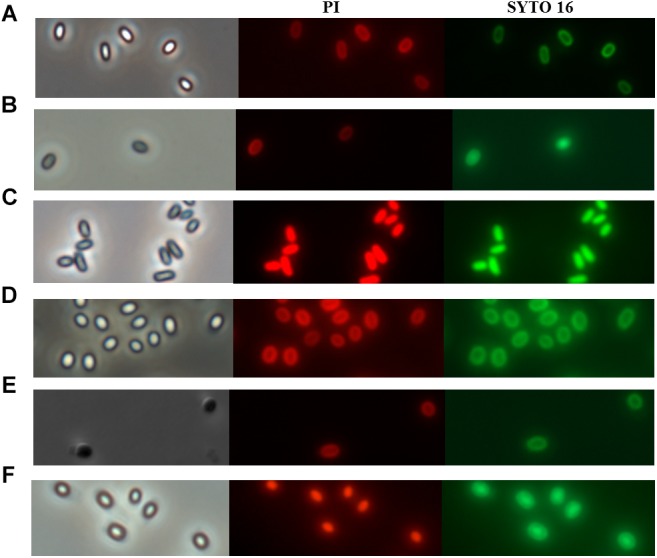
The phase contrast and fluorescence microscope of *B. subtilis*168 **(A–C)** and *C. sporogenes* PA3679 **(D–F)** spores: **(A,D)** untreated spores, **(C,F)** 600 MPa at 121°C for 1 min, **(B)** 600 MPa at ambient temperature for 10 min, **(E)** Incubation in 20 mM Tris buffer pH 7.4, L-alanine (100 mM), L-lactate (50 mM), and NaHCO_3_ at 30°C for 1 h.

Nowadays, the discussion about the mechanism of spore inactivation by PATS is mainly focused on whether spores undergo germination during inactivation by PATS treatment (Reineke et al., 2013b). Similarly, whether spores undergo germination during PAUHT treatment is the key point of figuring out how PAUHT inactivates them. In order to understand the mechanism of spore inactivation during PAUHT treatments, the structure changes of the *B. subtilis* 168 and *C. sporogenes* PA3679 spores during the process were investigated by staining the spores with PI and SYTO 16 after treatment, followed by observation using phase contrast microscopy and fluorescence microscopy. As shown in Figure 3, after PAUHT process, both *B. subtilis* 168 and *C. sporogenes* PA3679 spores turned from bright to gray, and could be stained by PI and SYTO 16 (Figure 3C,F). As PI and SYTO 16 staining were usually considered as the indicator of the integrity of spore’s IM and cortex (Mathys et al., 2007; Kong et al., 2010), respectively, we could ensure that the PAUHT treated spores displayed damaged IM and cortex. Moreover, more than 90% DPA released after the PAUHT treatment (Table 2), further confirming that the IM was damaged (Reineke et al., 2013a). However, we noticed that PAUHT treated spores turned gray rather than dark, indicating that the cores of these spores were only partially hydrated (Katja et al., 2013). Since the cortex played a key role in maintaining the core rehydration and germinated spores would degrade their cortex and turn dark (Figure 3B,E), it could be concluded that the cortex of PAUHT treated spores were only partially damaged. From these results, we could conclude that PAUHT-inactivated spores had damaged IM and partially damaged cortex, but we couldn’t tell whether spores went through germination because (1) heat-inactivated spores had a damaged IM and partially damaged cortex similar to PAUHT-inactivated spores, but didn’t go through germination (Setlow, 2006), and (2) spores that went through stage I of germination but didn’t accomplish the stage II of germination could be inactivated by PAUHT, and also exhibited damaged IM and partially damaged cortex.

**FIGURE 4:**
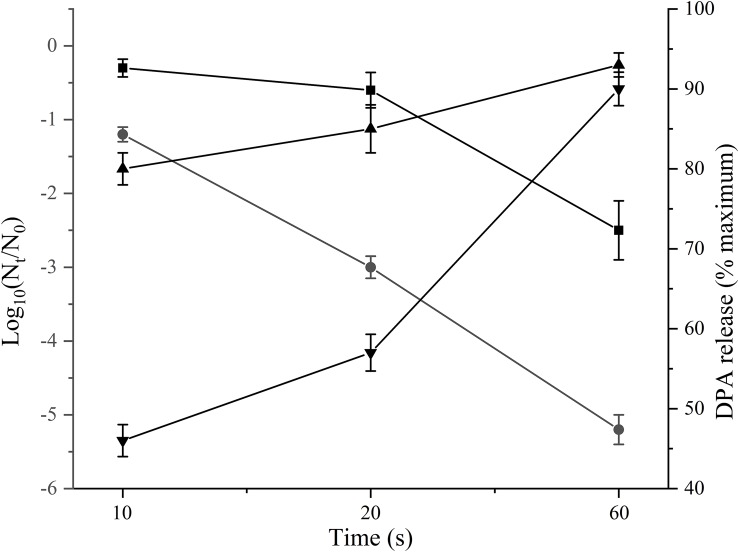
The inactivation of *B. subtilis* 168 (•) and *C. sporogenes* PA3679 spores (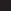) by thermal treatment at 121°C for different times, and its correlating amount of released DPA for *B. subtilis* 168 spores and (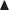) for *C. sporogenes* PA3679 spores (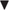), respectively.

**FIGURE 5:**
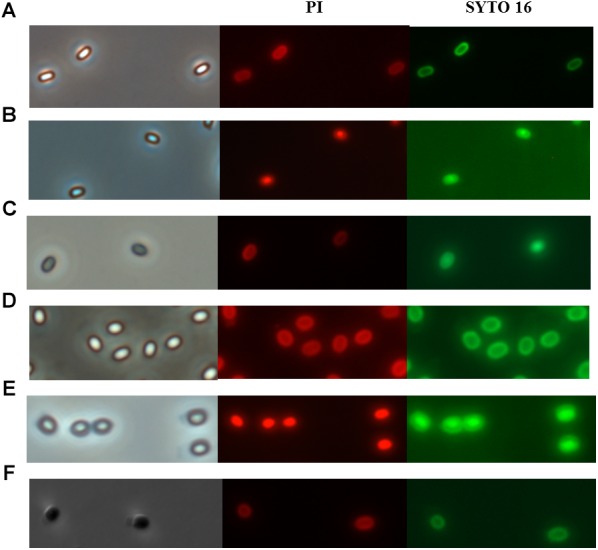
The phase contrast and fluorescence microscopy of *B. subtilis* 168 **(A–C)** and *C. sporogenes* PA3679 **(D–F)** spores: **(A,D)** untreated spores, **(B,E)** 121°C for 1 min at ambient pressure. **(C)** 600 MPa at ambient temperature for 10 min used as control, **(F)** Incubation in 20 mM Tris buffer pH 7.4, L-alanine (100 mM), L-lactate (50 mM), and NaHCO_3_ at 30°C for 1 h used as control.

Since the temperature during PAUHT was relatively high and it could play an important role in inactivation spores, we investigated the effect of heat treatment at temperatures same to PAUHT on spore inactivation. *B. subtilis* 168 and *C. sporogenes* PA3679 spores were treated at ambient pressure at 121°C for different times (10, 20, and 60 s), then the spores were stained by PI and SYTO 16 and observed by microscopy as described above. Thermal treatment of 121°C at 0.1 MPa for 1 min could induce 5.23 log and 2.51 log inactivation and DPA release (more than 90%) of *B. subtilis* 168 and *C. sporogenes* PA 3679 spores, respectively (Figure 4), indicating the temperature played an important role in spore inactivation during pressure holding time of PAUHT treatment. Moreover, compared to the untreated (Figure 5A,D) and germinated spores (Figure 5C,F), these heat treated spores were stained by both PI and SYTO 16 (Figure 5B,E), indicating the spore’s IM and cortex were damaged as previously studied (Setlow et al., 2008). Besides, the thermal treated spores turned from bright (Figure 5A,D) to gray (Figure 5B,E) rather than dark (Figure 5C,F) in the phase contrast microscopy, indicating the spore core was partially rehydrated, which is probably due to partially damage of cortex (Luu-Thi et al., 2015). These results were similar with the PAUHT treated spores, but we still could not confirm that the spores whether germinated during PAUHT treatment, because more than 90% DPA of these spores was released (Table 2), which could also be the results from germination by HP during PAUHT treatment.

**FIGURE 6:**
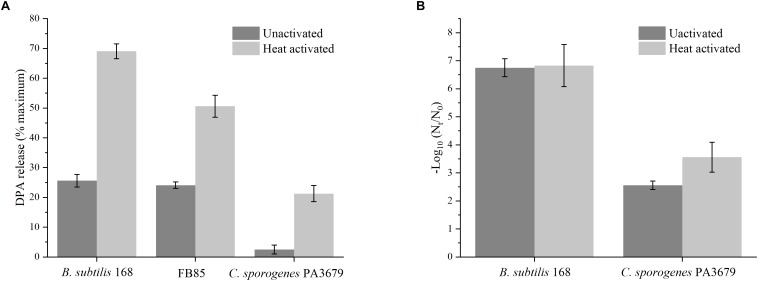
The DPA release or inactivation of unactivated and heat activated (75°C, 15 min) spores after treatment of 600 MPa at ambient temperature for 1 min **(A)** or PAUHT treatment of 600 MPa at 121°C without pressure holding time **(B)**.

In order to further investigate whether spores underwent germination during PAUHT treatment, we investigated the effect of pressure treatment alone on spore germination. The pressure treatment was performed at 600 MPa at ambient temperature for 1 min of holding time. The DPA release was determined after high pressure treatment and used as the indicator for spore germination as other reports (Yi and Setlow, 2010). As shown in Figure 6A, pressure at 600 MPa for 1 min could not directly inactivate the *B. subtilis* 168 and FB 85 spores (data was not shown), but it could induce more than 25% DPA to release, similar as previous studies (Vercammen et al., 2012; Sarker et al., 2015), indicating spores were induced to germinate by HP of 600 MPa. Since FB85 spores has no GRs (Paidhungat et al., 2002), thus germination under pressure of 600 MPa were likely triggered by opening the DPA channel without activating the GRs, as reported previously (Black et al., 2005; Doona et al., 2014). For *C. sporogenes* PA3679 spores. Pressure at 600 MPa for 1 min couldn’t induce spores to release DPA (Figure 6A), indicating *C. sporogenes* PA3679 spores could not germinate at this pressure. However, in the presence of SpoVA channels, the treatment of 600 MPa can theoretically induce *C. sporogenes* PA3679 spores to germinate similar to the *C. perfringens* spores (Paredes-Sabja et al., 2011; Doona et al., 2016b). It has been reported that the HP of 550 MPa triggered the *C. perfringens* spores to release their DPA and germinate, which happened after the cortex hydrolysis caused by activating the CLEs, SleC (Paredes-Sabja et al., 2009; Doona et al., 2016b). The *C. sporogenes* spores does not have the CSP and SleC (Paredes-Sabja et al., 2011; Doona et al., 2014), which are nececery for the cortex degradation of *C. perfringens* spores. Hence, the lack of CSP and SleC was probably the reason why HP at ambient temperature could not induce *C. sporogenes* PA 3679 spores to release DPA and germinate. In addition, previous reports indiccated that spore germination could be potentiated or activated by heat activation (Luu et al., 2015), hence we further investegate the effect of heat activation on spore germination triggered by HP (Figure 6A). Both *B. subtilis* 168 and *C. sporogenes* PA3679 spores were heat activated at 75°C for 15 min prior HP treatment of 600 MPa at atmosphere for 1 min. for *B. subtilis* 168 spores, heat activated spores released DPA (68%) more than unactivated spores (26%) (Figure 6A), indicating HP triggered germination was enhanced by heat activation as previous reports (Luu et al., 2015; Doona et al., 2016b). Notably, for *C. sporogenes* PA3679 spores, after the heat activation (75°C, 15 min), more than 20% DPA was released (Figure 6A), indicating the *C. sporogenes* PA3679 spores germinated after the HP of 600 MPa for 1 min. The effect of heat activation on *C. sporogenes* PA3679 spore germination by HP was surprising, since this has not been reported previously.

Considering the spore germination triggered by HP could be enhanced by heat activation, we further investigate the effect of heat activation on spore inactivation during PAUHT treatment. Both *B. subtilis* 168 and *C. sporogenes* PA3679 spores were heat activated at 75°C for 15 min prior PAUHT treatment of 600 MPa at 121°C without pressure holding time. After PAUHT treatment, the counts of spore inactivation were determined, as shown in Figure 6B. For *B. subtilis* 168 after heat activation, 6.83 log spores were inactivated by the compression during PAUHT treatment of 600 MPa at 121°C, which was comparative to unactivated spores (6.75 log inactivation), indicating that the heat activation has no effect on inactivation of *B. subtilis* 168 spores by PAUHT treatment. It could be attributed to the lack of GRs in *B. subtilis* 168 spores, since heat activation acted primarily on GRs (Luu et al., 2015). However, for *C. sporogenes* PA3679, 3.56 log of heat activated spores were inactivated by the same treatment, which was higher than unactivated spores (2.56 log inactivation), indicating that heat activation could significantly enhance the spore inactivation during compression of PAUHT treatment. These observations suggested that heat activation play different role on inactivation of different spores during compression. For *C. sporogenes* PA3679 spores, since the heat activation could not inactivate spores, but could enhance the spore germination by HP. Therefore, more heat activated spores were inactivated during compression, possibly because the HP triggered germination was enhanced by heat activation. Hence, the *C. sporogenes* PA3679 spores were probably triggered to germinate and then inactivated during compression of PAUHT treatment.

In order to further investigate whether spores undergo germination during compression of PAUHT treatment, the *B. subtilis* 168 and *C. sporogenes* PA3679 spores were treated with various conditions of *T_i_* = 65–84°C and high pressure *P* = 500–600 MPa during compression (hold time = 0 s). At the completion of the compression (100–120 s), the pressure was released, samples were withdrawn from the HPP chamber, cooled on ice, and then recovered on LB and RCM agar. As shown in Table 3. During compression of the PAUHT treatment of 600 MPa at 121°C (*T_i_* = 84 and *P* = 600 MPa), no increase of *B. subtilis* 168 and *C. sporogenes* PA3679 counts was discernible as above noted (Table 2), indicating the spores were not activated during compression of PAUHT treatment. Similar results were obtained for *B. subtilis* 168 spores with *T_i_* = 65–75°C and *P* = 500–600 MPa. However, for the *C. sporogenes* PA3679 spores, under the conditions of *T_i_* = 65°C and *P* = 600 MPa, the viable counts increased after the compression (98% increase), indicating the *C. sporogenes* PA3679 spores were activated after compression. At *T_i_* = 75°C and *P* = 600 MPa, a slight decrease (27%) in spore counts after the compression suggested that inactivation was preceded by activation. Hence, during compression of the PAUHT treatment (*T_i_* = 84°C and *P* = 600 MPa), the *C. sporogenes* PA3679 spores may also pass through the process of activation and inactivation.

**Table 3:** Activation and germination and of *B. subtilis* 168 and *C. sporogenes* PA3679 spores during compression.

*T_i_* (°C)	P (MPa)	Compression time (s)	Change%	
			
			*B. subtilis* 168	*C. sporogenes* PA3679
65	600	120	-45.7	+98.5
			-96.5^a^	-71.4^a^
75	500	100	-64.3	+65.2
			-91.3^a^	-53.7^a^
75	600	120	-75.2	-26.8
			-53.6^a^	-45.2^a^
84	600	120	-99.9	-99.7
			-5.8^a^	-9.1^a^


*Activation of spores was measured after compression, as increases in viable counts before and after compression. “^*a*^” represents spore germination. After compression, spores were subsequently treated by wet heat (80°C for 20 min) and then counted. The increase of viable counts before and after the wet heat treatment represents the germinated spores. All viability measurements are mean plate counts in triplicate.*

The effect of compression (*T_i_* = 65–84°C, *P* = 500–600 MPa) on the germination of *B. subtilis* 168 and *C. sporogenes* PA3679 spores were further investigated. After the completion of compression, spores were treated by wet heat (80°C, 20 min) then enumerated. As shown in Table 3. At *T_i_* = 75°C and *P* = 500 MPa or *T_i_* = 65°C and *P* = 600 MPa, for *B. subtilis* 168, more than 90% spores were inactivated by subsequent wet heat treatment, indicating the spores were induced to germinate by compression. At *T_i_* = 75°C and *P* = 600 MPa, the rate of germination decreased to 53% after compression, indicating some population of germinated spores were inactivated during compression. Similar results were obtained for *C. sporogenes* PA3679 spores. Hence, during compression of PAUHT treatment (*T_i_* = 84°C and *P* = 600 MPa), both *B. subtilis* 168 and *C. sporogenes* PA3679 spores possibly underwent the process of germination and inactivation.

As a consequence, during the PAUHT treatment, the compression and pressure holding process played important role in spore inactivation. The mechanism of spore inactivation during PAUHT treatment could be concluded as follows: for *B. subtilis* 168, spores were firstly induced to germinate under HP, which was enhanced by increased temperature. Then the germinated spores were inactivated by heat as previous reports (Luu et al., 2015; Doona et al., 2016a, 2017). For *C. sporogenes* PA3679, spores were likely activated during the compression time of PAUHT, and then the activated spores were induced to germination by HP, followed by inactivation by heat. In addition, the PAUHT inactivated spores showed gray rather than dark observed by phase contrast microscopy, indicating that these spores only undergo the first stage of germination (DPA was released and spores lost partial resistance) and the cortex degradation was blocked, which was possibly due to the inactivation of CLEs by heat or pressure (Reineke et al., 2011).

## Conclusion

In this work, PAUHT system was established by using soybean oil as adiabatic medium, and a regression model was developed to predict the initial temperature of soybean oil for PAUHT. The *B. subtilis* 168 and *C. sporogenes* PA3679 spores were inactivated during PAUHT treatment in short time (< 1 min). The inactivation mechanism of *B. subtilis 168* and *C. sporogenes* PA 3679 spores by PAUHT treatment could be supposed as follows: during PAUHT treatment, spores were firstly triggered to germinate by HP, with completing stage I of germination, and then the germinated spores were inactivated by heat.

## Industrial Relevance

The inactivation of bacterial spores in the low-acid canned food remains the most serious problem in food sterilization because of the high resistance of spores to high pressure and thermal processing. In this work, we showed that the proposed PAUHT system with soybean oil as adiabatic medium was effective for inactivating bacterial spores in short time (<1 min), therefore it has the potential to be a promising alternative technique for food sterilization. Further understanding of spore inactivation mechanism by PAUHT treatment will help to make this technique available for studies in pilot and production scale.

## Author Contributions

DL carried out the experiments and wrote the manuscript. LZ, XW, and PW gave the advice and assistance during the experiments. XL and FC reviewed the manuscript and gave the advice on the manuscript. XMW revised the manuscript. XH designed the experiments and reviewed the manuscript.

## Conflict of Interest Statement

The authors declare that the research was conducted in the absence of any commercial or financial relationships that could be construed as a potential conflict of interest.
