# Supplementary material for: Building of Pressure-Assisted Ultra-High Temperature System and Its Inactivation of Bacterial Spores
Source: Front Microbiol. 2019 Jun 10;10:1275. doi: 10.3389/fmicb.2019.01275 (PMC6579918; doi:10.3389/fmicb.2019.01275)
Supplement: Supplementary file 1 [file Data_Sheet_1.docx]

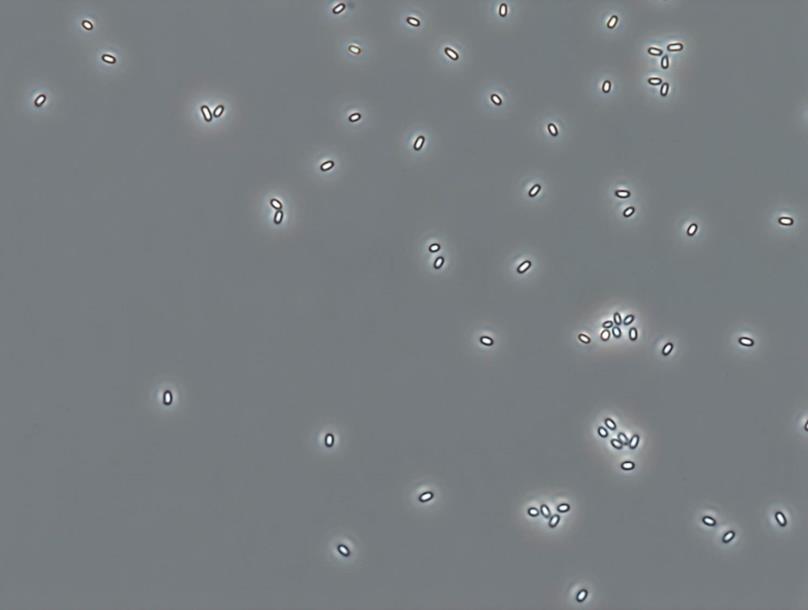


*B. subtilis* 168 spores


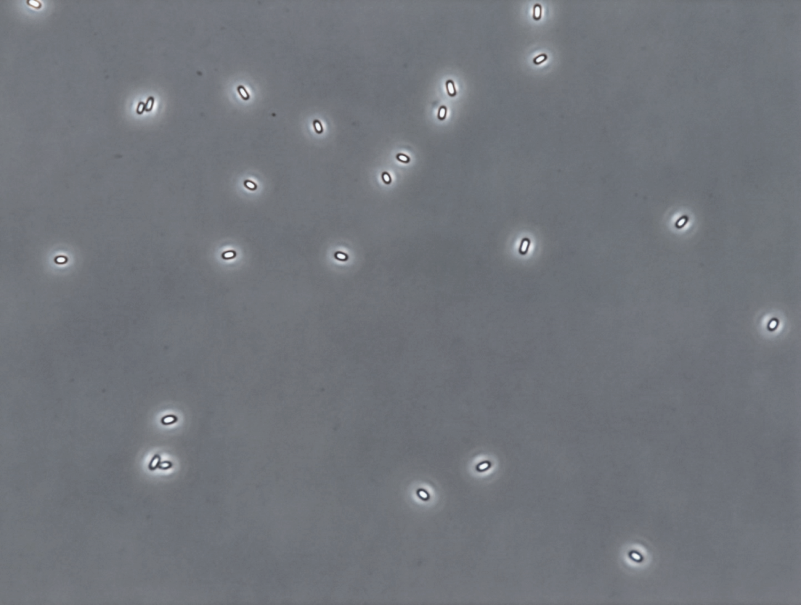


FB 85 (ΔGer ABKD) spores


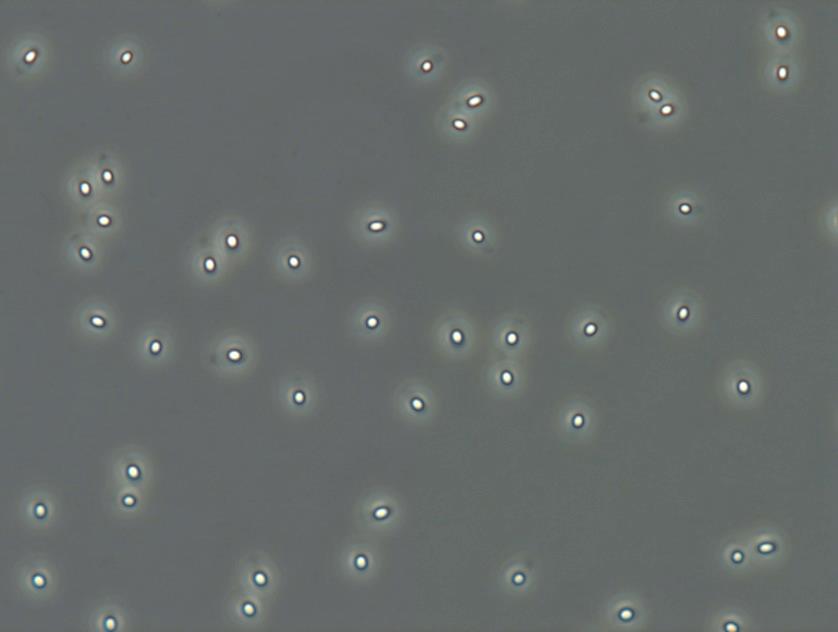


*C. sporogenes* PA3679 spores

Figure S1. The images of prepared spores observed by phase contrast microscopy
